# Genome-wide identification and analysis of the *EIN3/EIL* gene family in broomcorn millet (*Panicum miliaceum* L.)

**DOI:** 10.3389/fpls.2024.1440872

**Published:** 2024-08-07

**Authors:** Mengke Yang, Xiaohan Song, Jiazhen Li, Shiji Wang, Meichun Zhang, Xin Deng, Hongyan Wang

**Affiliations:** Laboratory of Plant Epigenetics and Evolution, School of Life Sciences, Liaoning University, Shenyang, China

**Keywords:** broomcorn millet, *EIN3/EIL*, phylogenetic evolution, conserved domain, cis-acting element, tissue-specific expression

## Abstract

The *EIN3/EIL* gene family holds a pivotal role as it encodes a crucial transcription factor in plants. During the process of polyploidization in broomcorn millet (*Panicum miliaceum* L.), there is an intriguing above-average amplification observed within the *EIN3/EIL* gene family. Nonetheless, our current knowledge of this gene family in broomcorn millet remains limited. Hence, in this study, we conducted a comprehensive analysis of the *EIN3/EIL* gene family in broomcorn millet, aiming to provide a deeper understanding of the potential evolutionary changes. Additionally, we analyzed the *EIN3/EIL* gene family of *Panicum hallii L*., a close relative of broomcorn millet, to enhance our characterization efforts. Within this study, we identified a total of 15 *EIN3/EIL* genes specific to broomcorn millet. Through covariance analysis, it was revealed that all *PmEIL* genes, except *PmEIL1* and *PmEIL15*, had duplicate copies generated through genome-wide duplication events. Importantly, the Ka/Ks values of all duplicated genes were found to be less than 1, indicating strong purifying selection. Phylogenetic analysis showed that these genes could be categorized into four distinct evolutionary branches, showcasing similar characteristics among members within the same branch. However, there appeared to be an uneven distribution of cis-acting elements amid the *EIN3/EIL* genes. Further examination of transcriptomic data shed light on the diverse spatiotemporal and stress-related expression patterns exhibited by the *EIN3/EIL* genes in broomcorn millet. Notably, under cold stress, the expression of *PmEIL3/4/8/14* was significantly up-regulated, while under drought stress, *PmEIL4/5/6* displayed significant up-regulation. Intriguingly, the expression pattern of *PmEIL15* showed an opposite pattern in resistant and sensitive cultivars. The findings of this study augment our understanding of the *EIN3/EIL* gene family in broomcorn millet and offer a valuable reference for future investigations into polyploid studies. Moreover, this study establishes a theoretical foundation for further exploration of the ethylene signaling pathway in broomcorn millet.

## 1 Introduction

Polyploidization constitutes a significant mechanism in the formation of angiosperm species, and all angiosperms experience at least one instance of polyploidization (Adams and Wendel, 2005). During this process, newly formed polyploids undergo extensive genetic modifications, granting them considerable evolutionary advantages to better adapt to environmental fluctuation (Ren et al., 2018). *Panicum miliaceum* L. is an interesting cereal crop of which the genome undergone hetero-tetraploidization approximately 5.8 million years ago, and coinciding with a pronounced expansion of the *EIN3/EIL* gene family (Zou et al., 2019).

The *EIN3/EIL* genes, decoding Ethylene Insensitive 3 (EIN3) and Ethylene Insensitive 3-like (EIL), form a plant-specific transcription factor family that serves as crucial regulators of ethylene signaling. They exert broad control over various aspects of plant growth, development, and defense against diverse stresses (Chen et al., 2005). Ethylene (ET), a vital gaseous hormone in plants, governs integral physiological processes such as plant growth, development, and senescence (Lee and Kim, 2003). To date, extensive investigations have elucidated the functions and characteristics of various *EIN3/EIL* genes in several plants, including Arabidopsis, tobacco, and rice (Mao et al., 2006; Hiraga et al., 2009; Wawrzynska et al., 2010). Notably, *OsEIL1* in rice plays a crucial role in wound signaling by binding to specific DNA sequences induced by trauma (Hiraga et al., 2009). Similarly, NtEIL2 in tobacco serves as a vital regulator of sulfur-induced gene expression, paralleling its homologous counterpart, AtSLIM1, which directly controls the expression of genes induced by sulfur starvation in tobacco (Wawrzynska et al., 2010).

Broomcorn millet is a C4 photosynthesis plant and closely related to bioenergy crops *Panicum virgatum* and *Panicum hallii*. Widely cultivated across semi-arid regions of Asia, Europe, and other continents, broomcorn millet boasts a remarkable history of global agrarian practices Barton (Adams and Wendel, 2005; Washburn et al., 2015; Zou et al., 2019). Its high-water use efficiency distinguishes it as an exceptionally drought-tolerant cereal crop, frequently employed in dryland agriculture where other crops struggle to flourish (Barton et al., 2009). Furthermore, broomcorn millet grains exhibit gluten-free properties, excellent nutritional content, and superior levels of proteins, minerals, and antioxidants compared to most other grains (Habiyaremye et al., 2017). Consequently, broomcorn millet holds promise as a source of human food security and offers prospects for a healthier diet in the face of the momentous climate changes occurring worldwide.

These excellent agronomic traits of broomcorn millet are presumably associated with its distinctive evolutionary process and genome components. *EIN3/EILs*, a family of transcription factors exclusive to plants, is hypothesized to have influence on the evolution of broomcorn millet (Zou et al., 2019). However, a comprehensive analysis specifically focusing on the *EIN3/EIL* gene family in broomcorn millet is still scarce. Therefore, it is very important to conduct a genome-wide identification and analysis of the potential function of *EIN3/EIL* genes. In this study, we systematically analyzed the *EIN3/EIL* gene family in broomcorn millet and *Panicum hallii* L., a close relative of broomcorn millet, to enhance our characterization efforts. We applied methodologies such as phylogenetic relationships, gene structure, and motif composition features to identify and classify *EIN3/EIL* genes. Furthermore, we conducted chromosomal localization, genome-wide covariance analysis, promoter cis-acting element identification, and expression profiling of the identified *EIN3/EIL* genes.

Overall, our findings establish a foundation for future functional investigations of the *EIN3/EIL* gene family in broomcorn millet, contributing significantly to our understanding of the ethylene signaling pathway within this particular species.

## 2 Materials and methods

### 2.1 Exploration, chromosomal localization and evolutionary analysis of the *EIN3/EIL* gene

The EIN3/EIL query sequences are 6 Arabidopsis EIN3/EIL protein sequences from TAIR (https://www.arabidopsis.org). The *Panicum miliaceum* genome was downloaded from NCBI (https://www.ncbi.nlm.nih.gov). The *Panicum hallii* genome was retrieved from the Phytozome (https://phytozome-next.jgi.doe.gov). All protein sequences were identified by CD-Search in NCBI with conserved domain (pfam04873). The BLAST was used to searched and identify the predicted broomcorn millet and *Panicum hallii EIN3/EIL* gene families using these confirmed sequences. *EIN3/EIL* genes with e values < 10^-10^ are considered candidate sequences, which were put into the pfam (pfam-legacy.xfam.org) and HMMER (hmmer.org) databases for validation of their conserved structural domains. The genomic information of broomcorn millet was used to locate the chromosomal location of the *EIN3/EIL* family genes by using the online software MapGene2Chrom (mg2c.iask.in/mg2c_v2.0) (Chao et al., 2015). The covariance results of broomcorn millet and *Panicum hallii EIN3/EIL* genes were obtained using the MCScanX function in TBtools, Ka/Ks values of CDS sequences were obtained using the Ka/Ks Calculator in TBtools, and the covariance relationships of *EIN3/EIL* genes were visualized using TBtools (Chen et al., 2020).

### 2.2 Phylogenetic analysis and characterization of EIN3/EIL proteins

The full amino acid sequences of EIN3/EIL members of these 6 different species were aligned using ClustalW. The EIN3/EIL protein sequences of Arabidopsis were obtained from TAIR and the other EIN3/EIL sequences of rice, *Panicum hallii*, *Setaria italica*, maize, and soybean were obtained from PlantTFDB (planttfdb.gao-lab.org). MEGA X software neighbor-joining method (NJ) was applied to construct a neighbor-joining phylogenetic tree, and the consistency of each node was evaluated using Bootstrap tests and 1000 replicates. The rest was carried out using the default parameters (Tamura et al., 2013). The results were embellished with iTOL (https://itol.embl.de). Molecular weights and isoelectric points of EIN3/EIL proteins were obtained from Expasy (https://www.expasy.org). Subcellular localization of EIN3/EIL proteins was analyzed using the Plant-mPLoc server (www.csbio.sjtu.edu.cn/bioinf/plant-multi) (Lescot et al., 2002). Ten conserved motifs of the EIN3/EIL proteins were identified using MEME (https://meme-suite.org/meme/doc/meme.html) and the results obtained were visualized using TBtools (Bailey et al., 2009; Chen et al., 2020).

### 2.3 Characterization of the *EIN3/EIL* gene family

*EIN3/EIL* gene structures were mapped using TBtools based on their genome annotations. The protein sequences of the broomcorn millet and *Panicum hallii EIN3/EIL* genes were uploaded to pfam and HMMER for analysis to obtain information on the conserved structural domains of the sequences. The 1.5 kb sequence upstream of the transcription start site (TSS) of the broomcorn millet and *Panicum hallii EIN3/EIL* genes was extracted and the PlantCARE (https://bioinformatics.psb.ugent.be/webtools/plantcare/html) database was used to predict cis-acting regulatory elements in the promoter region of the *EIN3/EIL* gene (1.5 kb upstream of the ATG start site), and the results obtained were visualized using TBtools (Chen et al., 2020).

### 2.4 Analysis of tissue-specific expression of *EIN3/EIL* genes and their response to abiotic stresses

Transcriptome data for analyzing the expression pattern of *EIN3/EIL* family genes in broomcorn millet were obtained at NCBI (BioProject: PRJNA431485). Transcriptome data for drought, cold, and salt stress in broomcorn millet were downloaded from NCBI (BioProject: PRJNA454008, PRJNA650146, PRJNA592319) (Meng et al., 2021; Yuan et al., 2021; Cao et al., 2022). The drought-tolerant variety of millet, NM5, and the sensitive variety, JS6, were treated with 20% PEG-6000 solution to simulate drought stress, and the leaves were taken for transcriptome sequencing at 6 and 24 h, respectively (Cao et al., 2022); broomcorn millet planted for 17 days was subjected to low temperature treatment at 6°C to simulate cold stress and seedlings were taken at 0.5, 1, 3, 6, 16 and 24 h for transcriptome sequencing (Meng et al., 2021); broomcorn millet salt-tolerant variety ST 47 and sensitive variety SS 212 were cultured in ½ Hoagland nutrient solution containing 1% (w/w) NaCl to mimic salt stress, and roots were taken for transcriptome sequencing at 0 h, 12 h, 1 d, 3 d, 7 d, and on day 7 of re-watering (RW 7 d) (Yuan et al., 2021). The expression pattern of *PmEIL* gene was analyzed under various abiotic stresses and heat maps were drawn using HeatMap tool of TBtools.

## 3 Results

### 3.1 Characterization and evolutionary analysis of *EIN3/EIL* genes

#### 3.1.1 Identification and chromosomal distribution of *EIN3/EIL* genes

**Figure 1:**
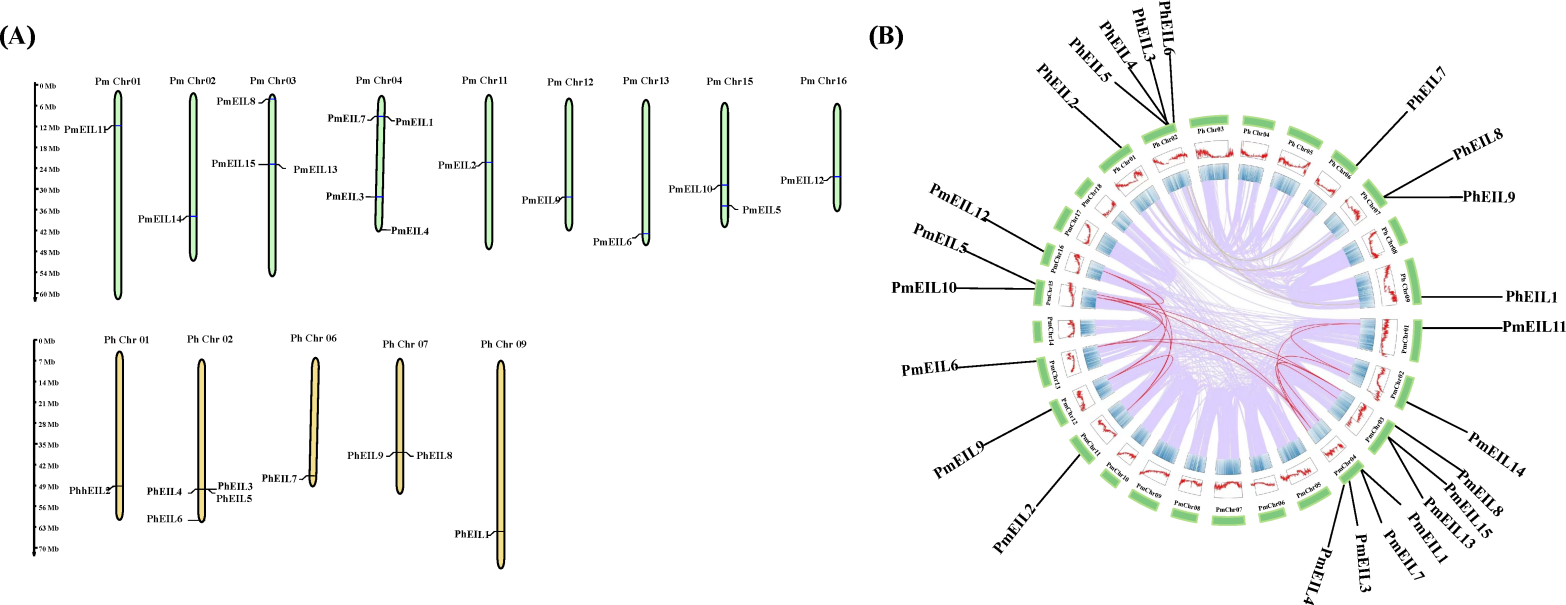
Chromosomal localization and genome-wide covariance analysis of *EIN3/EIL* genes in broomcorn millet and *Panicum hallii*. **(A)** Chromosomal localization of *EIN3/EIL* genes in broomcorn millet and *Panicum hallii*. The broomcorn millet *EIN3/EIL* genes are represented by *PmEIL1-PmEIL15* as shown in **Table 1**, and PmChr01-PmChr18 represent the 18 assembled chromosomes of broomcorn millet, respectively. *Panicum hallii EIN3/EIL* genes are represented by *PhEIL1-PhEIL9* as shown in **Table 1**, and PhChr01-PhChr09 represent the nine assembled chromosomes of the closely related species, respectively. **(B)** Genome-wide covariance information for broomcorn millet and closely related species. Genes and chromosomes are represented in the same way as in Figure **(A)**.

Using a homology-based method, we conducted a similarity search for genes in broomcorn millet and *Panicum hallii* to identify the *EIN3/EIL* genes. The query sequence was comprised of the concerved domain (pfam04873) and the six Arabidopsis EIN3/EIL protein sequences from TAIR. As a result, we identified a total of 15 *EIN3/EIL* genes in broomcorn millet and 9 in *Panicum hallii*. These genes were named as *PmEIL1* to *PmEIL15* and *PhEIL1* to *PhEIL9*, respectively (**Table 1**). By employing the MapInspector tool, we successfully mapped the identified *EIN3/EIL* genes to their respective chromosomal locations. The *EIN3/EIL* genes were found to be distributed across the assembled chromosomes (**Figure 1A**), exhibiting a heterogeneous pattern. For instance, Chr03, Chr04, and Chr15 harbored 3,4,2 *EIN3/EIL* genes in broomcorn millet, respectively. In contrast, Chr01-02, Chr11-13, and Chr16 contained only one *EIN3/EIL* gene each, while the remaining chromosomes lacked any *EIN3/EIL* distribution.

**Table 1:** Information on all *EIN3/EIL* genes identified in *broomcorn millet* and *Panicum hallii*.

name	EIL mRNA ID	EIL protein ID	Number of amino acid	Molecular weight Mw/Da	Theoretical pI	Number of negatively amino acid	Number of positively amino acid	Instability index	Aliphatic index	Grand average of hydropathicity (GRAVY)	Cellular localization
*PmEIL1*	PM04G07060	RLM86675.1	414	42332.04	5.81	36	29	46.88	85	0.005	Nucleus
*PmEIL2*	PM11G08880	RLN07303.1	431	47474.47	5.05	72	49	61.49	77.7	-0.546	Nucleus
*PmEIL3*	PM04G19830	RLM84513.1	610	68138.39	5.42	92	71	51.84	75.61	-0.726	Nucleus
*PmEIL4*	PM04G34370	RLM85795.1	597	64353.46	5.92	71	62	57.2	63.2	-0.515	Nucleus
*PmEIL5*	PM15G18500	RLM73422.1	641	70225.68	5.98	80	71	45.25	70.67	-0.643	Nucleus
*PmEIL6*	PM13G22070	RLN04204.1	652	71528.15	5.79	81	69	43.84	72.16	-0.625	Nucleus
*PmEIL7*	PM04G07050	RLM86272.1	502	52961.71	5.25	58	38	53.55	73.63	-0.225	Nucleus
*PmEIL8*	PM03G01550	RLN36231.1	610	68216.67	5.52	91	72	47.39	78.46	-0.690	Nucleus
*PmEIL9*	PM12G18190	RLM80807.1	439	47662.92	5.48	65	51	57.68	79.41	-0.445	Nucleus
*PmEIL10*	PM15G10390	RLM73531.1	516	56020.31	5.26	84	58	49.57	69.71	-0.673	Nucleus
*PmEIL11*	PM01G13700	RLN40963.1	643	71609.10	4.95	89	66	53.06	57.68	-0.773	Nucleus
*PmEIL12*	PM16G09610	RLM64998.1	316	34510.63	5.42	48	31	39.77	78.80	-0.516	Nucleus
*PmEIL13*	PM03G25020	RLN35715.1	367	37833.35	9.01	26	31	50.14	85.53	-0.035	Nucleus
*PmEIL14*	PM02G27460	RLN19248.1	644	71580.17	5.08	86	67	53.78	55.95	-0.774	Nucleus
*PmEIL15*	PM03G25030	RLN33384.1	650	69740.99	9.97	57	85	58.53	75.25	-0.356	Nucleus
*PhEIL1*	XM_025937033.1	XP_025792818.1	644	71559.1	5.05	87	67	52.93	56.24	-0.764	Nucleus
*PhEIL2*	XM_025941444.1	XP_025797229.1	467	50717.01	5.17	74	51	59.55	73.83	-0.533	Nucleus
*PhEIL3*	XM_025944855.1	XP_025800640.1	610	67949.29	5.62	89	72	50.15	77.21	-0.7	Nucleus
*PhEIL4*	XM_025944856.1	XP_025800641.1	608	67939.38	5.6	86	68	49.15	78.21	-0.689	Nucleus
*PhEIL5*	XM_025944857.1	XP_025800642.1	612	67969.29	5.62	89	74	50.15	77.51	-0.71	Nucleus
*PhEIL6*	XM_025946653.1	XP_025802438.1	605	65112.24	5.87	72	63	56.07	63.98	-0.514	Nucleus
*PhEIL7*	XM_025964396.1	XP_025820181.1	537	58584.54	5.93	61	54	45.72	68.18	-0.633	Nucleus
*PhEIL8*	XM_025967590.1	XP_025823375.1	512	56124.52	5.25	83	56	44.5	70.57	-0.674	Nucleus
*PhEIL9*	XM_025967591.1	XP_025823376.1	469	51392.18	5.43	74	52	46.26	67.27	-0.724	Nucleus

#### 3.1.2 Evolutionary analysis of *EIN3/EIL* genes

Given that broomcorn millet is a hetero-tetraploid plant that experienced whole-genome duplication (WGD), resulting in the amplification of the *EIN3/EIL* gene family (Zou et al., 2019), it becomes crucial to analyze the duplication status of the *EIN3/EIL* genes. Through covariance analysis, we revealed that all *PmEIL* genes, except *PmEIL1* and *PmEIL15*, possessed duplicate copies derived from the WGD events (**Figure 1B**). Furthermore, the duplication level of *PmEIL* genes was approximately five times higher than that of *PhEIL*s. Previous studies have indicated that synonymous mutations are not influenced by natural selection, whereas nonsynonymous mutations are subject to natural selection during evolution (Zhang et al., 2006). The ratio between the frequency of nonsynonymous substitutions (*Ka*) and the frequency of synonymous substitutions (*Ks*) is often used to gauge the selection pressure and evolutionary rate of duplicated gene pairs. A *Ka/Ks* value > 1 suggests positive selection, *Ka/Ks* < 1 indicates purifying selection, and *Ka/Ks* = 1 signifies neutral selection or no selection. In our analysis, we observed that the *Ka/Ks* values of all duplicated genes were < 1 (**Supplementary Table S1**), implying that all broomcorn millet *EIL* genes underwent purifying selection during evolution.

### 3.2 Phylogenetic analysis of EIN3/EIL proteins

**Figure 2:**
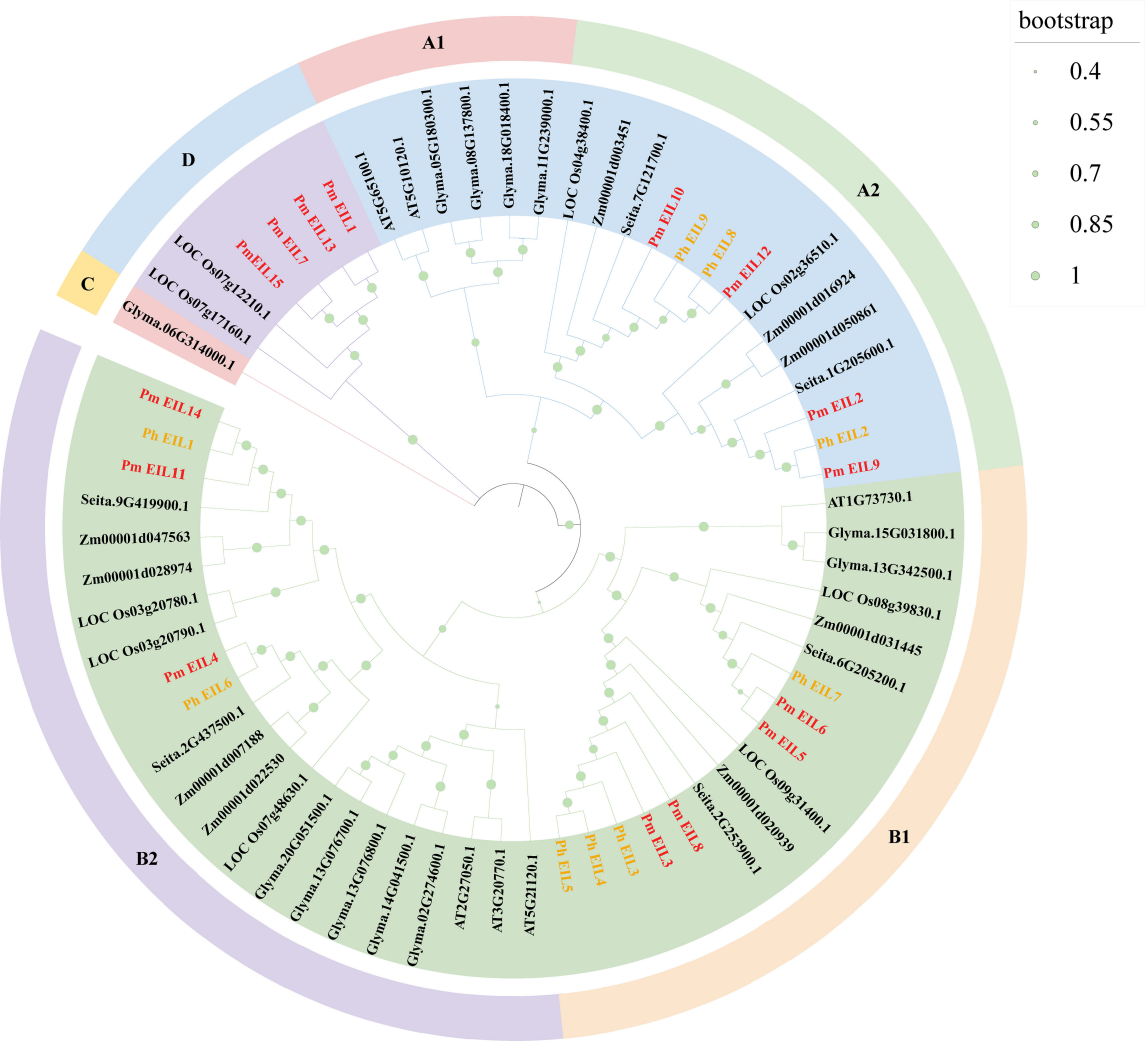
Phylogenetic tree of EIN3/EIL proteins in seven species. The symbols Pm, AT, LOC, Ph, Seita, Zm, and Glyma represent broomcorn millet, Arabidopsis, rice, Panicum hallii, Setaria italica, maize, and soybean, respectively. The inner circles are marked in blue, green, pink and purple, representing branches A, B, C and D, respectively. Each branch is further divided into subbranches and marked with different colors and symbols on the outer circle.

To investigate the functions of the members of EIN3/EIL protein family, we constructed a phylogenetic tree encompassing EIN3/EIL proteins from broomcorn millet and six other species, including wild relatives (**Figure 2**). The phylogenetic tree consisted of 60 EIN3/EIL proteins, which could be clearly categorized into four distinct evolutionary branches, denoted as A, B, C, and D. These branches further exhibited subdivision into six sub-evolutionary branches, namely A1, A2, B1, B2, C, and D.

Evolutionary branch A included PmEIL2, PmEIL9-10, and PmEIL12, while evolutionary branch B comprised PmEIL3-6, PmEIL8, PmEIL11, and PmEIL14. Evolutionary branch C was composed solely of a single soybean EIL protein (Glyma.06G314000.1). Lastly, evolutionary branch D consisted of PmEIL1, PmEIL7, PmEIL13, and PmEIL15. In this phylogenetic tree, EIN3/EIL proteins in monocotyledons and dicots primarily clustered within distinct sub-evolutionary branches. Dicotyledonous soybean’s EIN3/EIL proteins predominantly clustered in sub-branches A1, B2, and C, while those from monocotyledonous plants clustered in the remaining sub-branches.

Within the phylogenetic tree, evolutionary branch A encompassed 20 EIN3/EIL proteins, evolutionary branch B contained 39 EIN3/EIL proteins, evolutionary branch C consisted of one protein, and evolutionary branch D comprised six proteins. This observation highlights that the distribution of EIN3/EIL genes across the four evolutionary branches, A, B, C, and D, is not uniform.

### 3.3 Gene structure analysis of *EIN3/EIL* genes

**Figure 3:**
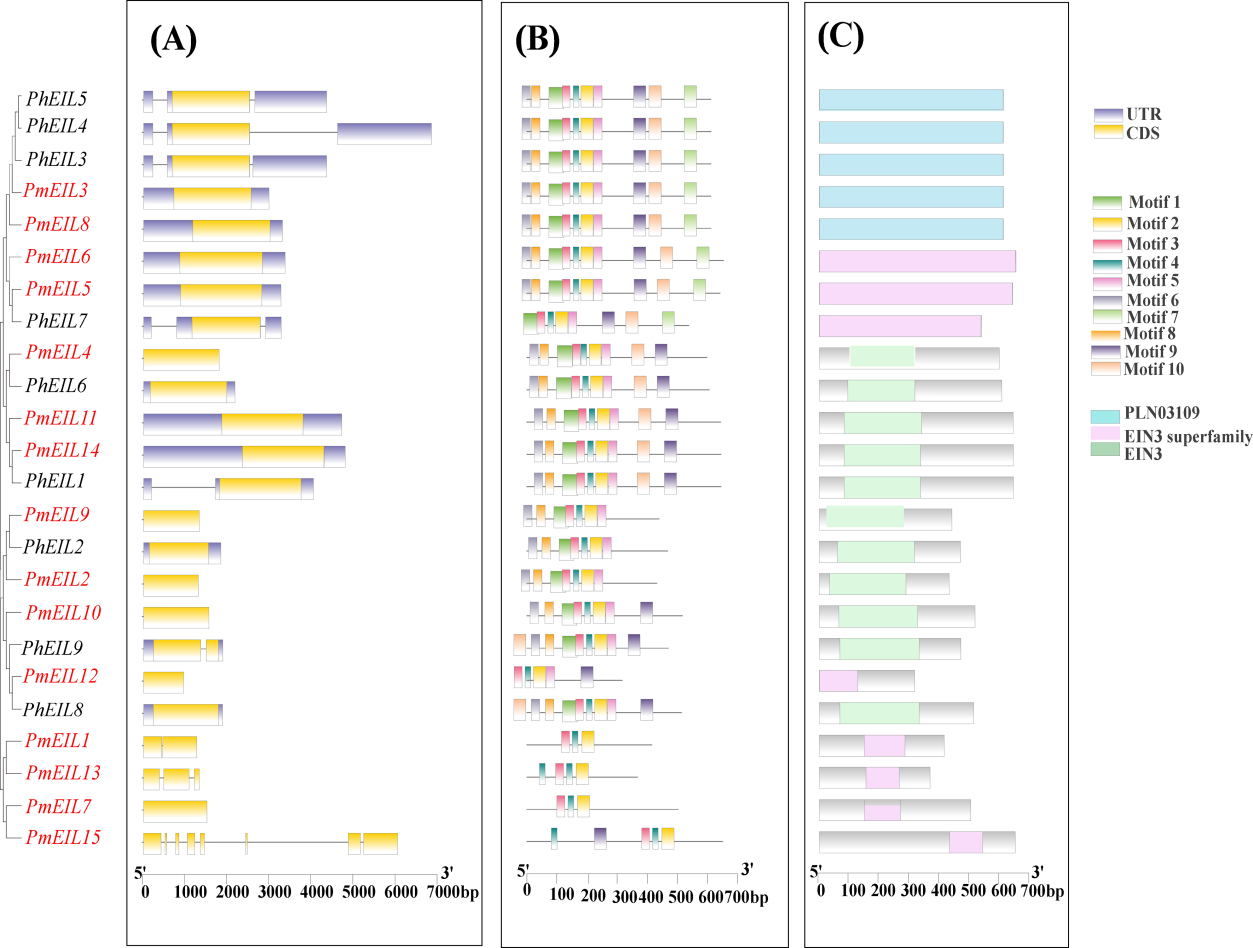
Gene structures, conserved motifs, and conserved structural domains of the *EIN3/EIL* genes in broomcorn millet and *Panicum hallii*. **(A)** Gene structures of *EIN3/EILs*; **(B)** Motif analysis of *EIN3/EILs* genes; **(C)** Conserved structural domains of *EIN3/EILs.*.

The diversity of gene structures serves as a significant resource for the evolution of gene families (Pellicer et al., 2018). To delve into the structural diversity of *EIN3/EIL* genes, we conducted an analysis of the exon-intron structures of the identified genes. As depicted in **Figure 3**, *EIN3/EIL* genes within the same phylogenetic group exhibited similar gene structures. Notably, 12 broomcorn millet *EIN3/EIL* genes displayed a loss of introns compared to those observed in *Panicum hallii*. These findings suggest that the broomcorn millet *EIN3/EIL* gene family underwent intron/exon acquisition or loss events during the course of evolution.

To further elucidate the characteristics of broomcorn millet *EIN3/EIL* genes, we examined ten conserved motifs within their protein sequences (**Figure 3B**). Our analysis revealed that the N-terminal motifs of broomcorn millet EIN3/EIL proteins exhibited a high degree of conservation, with the exception of PmEIL1, PmEIL13, PmEIL7, and PmEIL15, which did not display significant conservation in both N- and C- terminal motifs. In contrast to the highly conserved N-terminal motifs, the C-terminal motifs demonstrated a lesser degree of conservation. Furthermore, the conserved motifs of broomcorn millet *EIL* genes within the same phylogenetic group generally exhibited lower levels of conservation compared to those of *Panicum hallii*. The analysis of conserved domains shows that *PmEILs* and *PhEILs* contain the EIN3, EIN3 superfamily, and PLN03109 domains. The conserved domains of the same subfamily are highly similar (**Figure 3C**).

### 3.4 Analysis of *cis*-regulatory elements in the promoter region of *EIN3/EIL* genes

**Figure 4:**
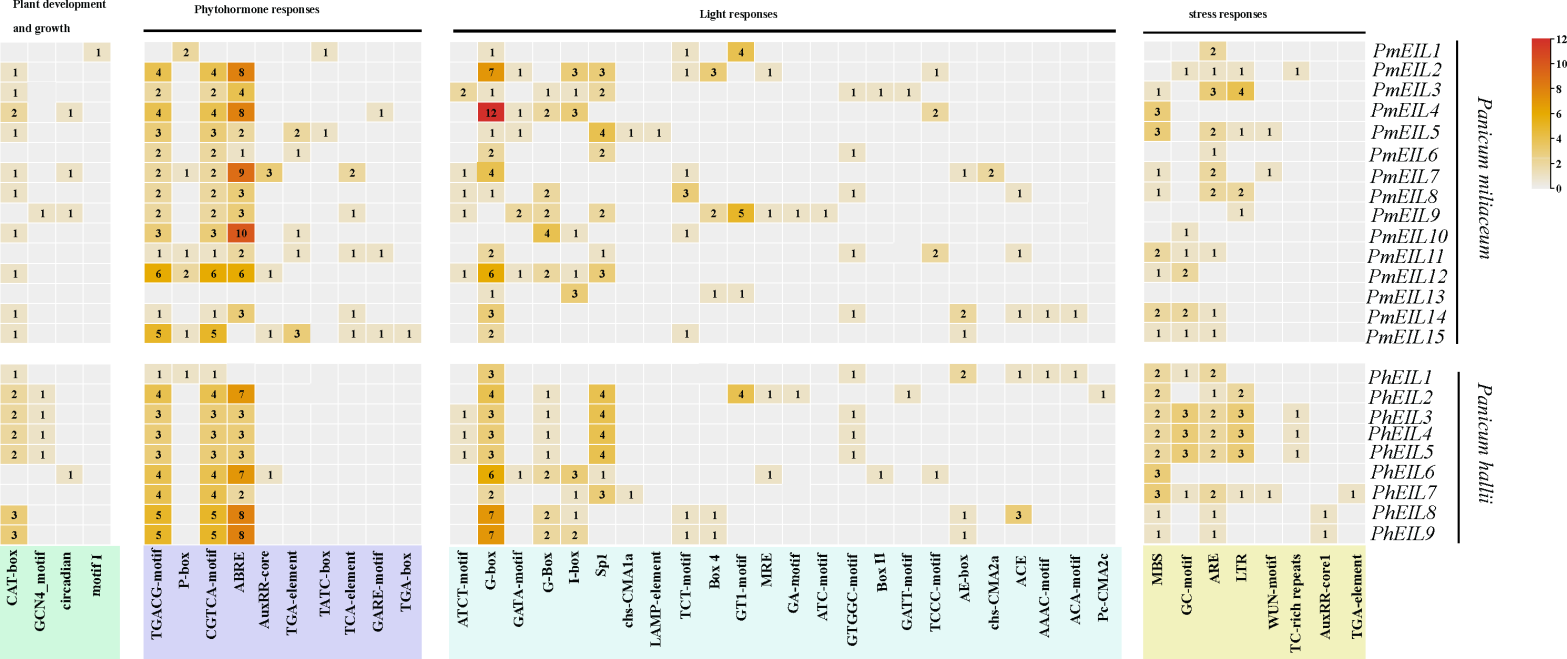
*Cis*-acting elements on the promoters of *EIN3/EIL* genes in broomcorn millet and *Panicum hallii*. *Pm* represents the *EIN3/EIL* gene of broomcorn millet and *Ph* represents the *EIN3/EIL* gene of *Panicum hallii*.

*cis*-Regulatory elements located in the promoter regions of genes play a crucial role in transcriptional regulation (Nuruzzaman et al., 2014). Our investigation focused on analyzing the *cis*-regulatory elements present in the promoters of *EIN3/E*IL genes in both broomcorn millet and *Panicum hallii*. The promoter regions of these *EIN3/EIL* genes exhibited a diverse array of *cis*-regulatory elements, as illustrated in **Figure 4**. We meticulously identified and quantified *cis*-regulatory elements associated with the regulation of plant growth and development, phytohormone responses, light responses, and stress responses in the promoters of all *EIN3/EIL* genes within broomcorn millet and *Panicum hallii*.

Among the identified *cis*-regulatory elements, four of them were specifically implicated in the growth and development of plants. For instance, the GCN4_motif is intricately involved in the expression of endosperm genes (Bhattacharyya et al., 2012). The CAT-box represents a *cis*-regulatory element associated with hyphal tissue expression (Anderson et al., 2003). The circadian element is associated with the regulation of circadian rhythm (Bobb et al., 1997), and there was a noteworthy increase in the number of circadian elements in broomcorn millet *EIN3/EIL* genes compared to *Panicum hallii*. Additionally, Motif I, which exclusively exists in *PmEIL1*, is associated with certain root-specific responses.

To elucidate the connection between *cis*-regulatory elements and phytohormone responses, we identified 10 *cis*-regulatory elements linked to various phytohormones. These include response elements for methyl jasmonate (MeJA), such as CGTCA-motif and TGACG-motif (Ulmasov et al., 1997); response elements for growth hormone (IAA), such as TGA-element and AuxRR-core (Kim et al., 1992); and response elements for gibberellin (GA), such as GARE-motif and P-box (Shen and Ho, 1995) Notably, the presence of ABREs (Abscisic Acid Responsive Elements) in *PmEIL2*, *PmEIL4*, *PmEIL7*, and *PmEIL10* was significantly more abundant compared to other *PmEIL*s and *PhEIL*s.

Remarkably, the TGA-element, TATC-box, TCA-element, GARE-motif and TGA-box were exclusively identified in broomcorn millet but absent in *Panicum hallii.* Furthermore, 8 *cis*-regulatory elements associated with stress responses. 24 cis-regulatory elements associated with light responsiveness, which indicated the majority cis-regulatory elements in the promoter regions of *EIN3/EIL* genes in broomcorn millet were primarily responsive to light stimuli.

### 3.5 Spatio-temporal expression profiling of *EIN3/EIL* genes

**Figure 5:**
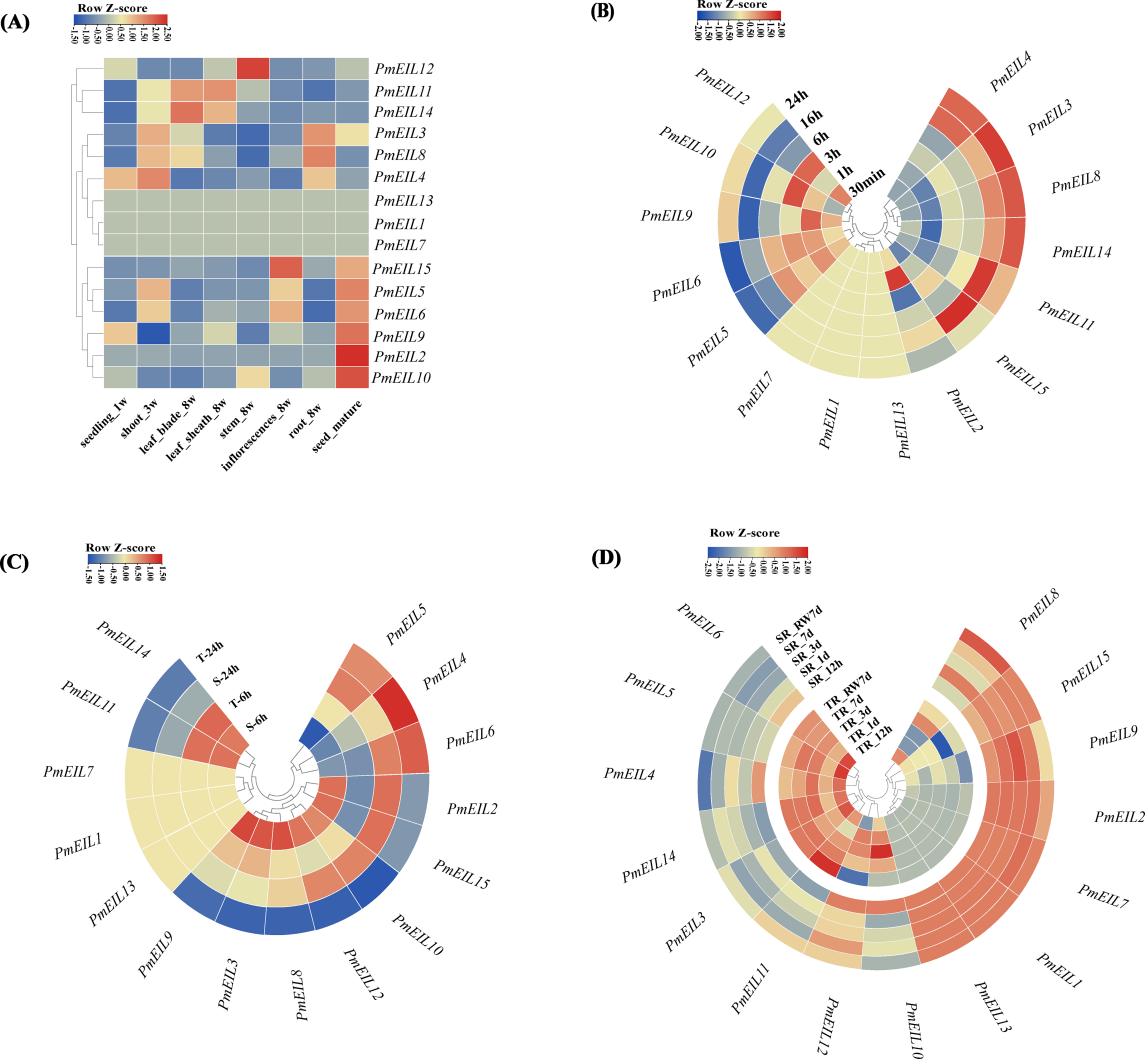
Spatio-temporal expression profile of *EIN3/EIL* genes under abiotic stresses in broomcorn millet. **(A)** Expression patterns of *PmEILs* in eight representative tissues (one-week germinated seeds, three-week seedlings, eight-week leaves, leaf sheaths, stems as well as inflorescences and roots, and mature seeds); **(B)** Expression patterns of *PmEILs* under cold stress; **(C)** Expression patterns of *PmEILs* under drought stress (T for tolerant varieties and S for sensitive varieties); **(D)** Expression patterns of *PmEILs* in roots under salt stress (RW for re-watering, TR for roots of tolerant varieties, SL for roots of sensitive varieties); **(B–D)** Gradient colors represent the expression of genes in different samples compared to the control group, as indicated by the z score of expression that is scaled across genes (Expression is scaled across genes) ^[35]^.

To gain insights into the expression profiles and potential biological functions of all *EIN3/EIL* genes in broomcorn millet, we conducted an analysis of their expression patterns using RNA-seq data obtained from NCBI. The data encompassed eight different tissues/organs of broomcorn millet at various growth and developmental stages. Overall, most of the *EIN3/EIL* genes exhibited expression in all eight tissues and organs (**Figure 5**). The expression levels of *PmEIL*s varied across tissues within the same growth period and across different growth periods within the same tissue (**Figure 5A**).

Among the identified genes, *PmEIL3*, *PmEIL4*, and *PmEIL8* displayed high expression levels in three-week-old shoots and eight-week-old roots compared to other tissues. *PmEIL12* exhibited marked expression in eight-week-old stems, while *PmEIL11* and *PmEIL14* demonstrated remarkably high expression in eight-week-old leaf blades and leaf sheaths (with RPKM values reaching up to 136.44 and 239.32 in leaves, and 139.85 and 197.85 in leaf sheaths, respectively), which suggested that these 6 *PmEILs* likely play a more prominent role in the vegetative growth period of broomcorn millet. Conversely, *PmEIL15*, *PmEIL5*, *PmEIL6*, *PmEIL9*, *PmEIL2*, and *PmEIL10* exhibited high expression levels in mature seeds but relatively lower expression in other vegetative organs, indicating that these genes may contribute to seed maturation. *PmEIL13*, *PmEIL1* and *PmEIL7* exhibited no expression across all analyzed tissues.

Overall, variations in the expression patterns of broomcorn millet’s *EIN3/EIL* genes were observed during different growth periods. Furthermore, genes exhibiting alterations in structural features and conserved motifs underwent significant changes in their expression levels, highlighting the complex regulatory mechanisms involved in the expression of these genes in broomcorn millet.

### 3.6 *EIN3/EIL* gene expression patterns under abiotic stresses

Ethylene, a crucial signaling molecule in plants, has been implicated in various stress responses. Among the ethylene signaling molecules, EIN3 and EIL1 have been shown to modulate stress responses in plants (Tao et al., 2015; Dubois et al., 2018). Of the diverse abiotic stresses that pose limitations to plant growth and development, soil drought, salt stress, and cold stress are widely recognized as common and severe stressors. To investigate the potential biological roles of *PmEIN3/EIL* genes in these stress conditions, we examined their expression patterns under salt (NaCl), drought, and cold treatments using previously published RNA-seq data (Meng et al., 2021; Yuan et al., 2021; Cao et al., 2022).

As depicted in **Figures 5B–D**, substantial distinctions in the stress-induced expression patterns of broomcorn millet’s *EIN3/EIL* genes were observed. For instance, *PmEIL1/7/13* exhibited low or negligible expression levels under each abiotic stress. Under cold stress (**Figure 5B**), *PmEIL3/4/8/14* gradually significant (p<0.05) upregulated as the duration of cold treatment increased compared to the control (0 h), with the peak upregulation occurring at 24 h of treatment. On the other hand, the expression of *PmEIL*5/6/9/10/12 was initially upregulated at the 3-hour time point before gradually downregulating, indicating their potential involvement in the transient response of broomcorn millet to cold stress.

Under drought stress (**Figure 5C**), *PmEIL11/14* exhibited an upregulation followed by downregulation, displaying a more pronounced response in resistant varieties. *PmEIL4/5/6* demonstrated significant (*p*<0.05) upregulation in both resistant and sensitive varieties as the treatment duration increased, while *PmEIL15* showed downregulation in resistant varieties and the opposite trend in sensitive varieties. In roots under salt stress (**Figure 5D**), notable variations were observed in the expression patterns between resistant and sensitive varieties. Specifically, *PmEIL3/4/5/6/10/11/12/14* exhibited significant (*p*<0.05) upregulation in resistant varieties, while either no significant difference or a downward trend was observed in sensitive varieties. Conversely, *PmEIL1/2/7/8/9/13/15* displayed no significant difference or a downward trend in resistant varieties but showed significant (*p*<0.05) upregulation in sensitive varieties.

These findings provide valuable insights into the expression patterns of *PmEIN3/EIL* genes under different stress conditions, shedding light on their potential roles in broomcorn millet’s response to abiotic stresses.

## 4 Discussion

Polyploidization is a recurrent occurrence in the evolutionary history of angiosperms plants, and it entails more than the simple addition of genetic material (Adams and Wendel, 2005). Broomcorn millet, compared to its close relative *Panicum hallii*, exhibited an above-average amplification of the *EIN3/EIL* gene family during polyploidization (Zou et al., 2019). Consequently, broomcorn millet presents itself as a promising model for studying the impact of polyploidy on the *EIN3/EI*L gene family. The *EIN3/EIL* genes are known to play a crucial role in the ethylene signaling pathway and serve as key integrators of ethylene signals along with other signaling pathways, thereby influencing the regulation of plant growth and development (Singh et al., 1992; Chen et al., 2005; Wawrzynska and Sirko, 2014). While the regulatory mechanisms of *EIN3/EIL* genes have been extensively studied in Arabidopsis, tobacco, and rice, their functions in broomcorn millet remain unclear. Therefore, our objective was to identify and analyze the *EIN3/EIL* gene family in heterozygous tetraploid broomcorn millet, aiming to unravel the role of this gene family in the natural formation of broomcorn millet. In this study, we provided a comprehensive characterization of the broomcorn millet *EIN3/EIL* gene family. It is important to remark that the identified *EIN3/EIL* genes were obtained from a representative genome assembly, which might not encompass all four sets of genes present in the species. Consequently, the genes identified in this study serve as representative sequences, and their authenticity should be confirmed in future experimental validations.

### 4.1 The *EIN3/EIL* genes acquired duplicate copies during polyploidization and were subjected to purifying selection

Polyploidization can lead to gene duplication that increased potential for evolutionary adaptation and surviving environmental turmoil (Barton et al., 2009; Ebadi et al., 2023). Covariance analysis revealed that the majority of *PmEIL* genes (13) possessed duplicate copies resulting from genome-wide replication events. Considering the potential advantageous effects of duplicated genes on the organism, we hypothesize that this amplification may enhance broomcorn millet’s ability to adapt to arid and semi-arid climates. Moreover, we observed that *PmEIL1* and *PmEIL7*, as well as *PmEIL13* and *PmEIL15*, were located adjacently on the same chromosome. This suggests that they were from tandem duplication. These patterns lead us to speculate that evolutionary constraints have contributed to the functional stability of these *PmEIL* genes, thereby facilitating its crucial role during critical growth and developmental stages in broomcorn millet.

### 4.2 The structural characteristics of *EIN3/EIL* genes and their association with expression levels in broomcorn millet

Previous research showed introns can exert an influence on gene transcription levels by modulating transcription rates, nuclear translocation efficiency, and transcript stability (Shaul, 2017). It was observed that 12 *EIN3/EIL* genes had no intron in broomcorn millet compared to *Panicum hallii* (**Figure 3A**). This implies that intron loss occurred in the *EIN3/EIL* gene family of broomcorn millet during polyploidization. Analysis of conserved motifs suggests that the C-terminal motifs may serve as the primary source of variation among EIN3/EIL family members. The motifs of *PmEIL1*, *PmEIL13* and *PmEIL7* exhibited limited conservation in both N-terminal and C-terminal motifs (**Figure 3B**). Notably, those three *EIN3/EIL* genes showed very lower expression level in organs at different growth stages and abiotic stresses (**Figure 5**). Corroborating these findings, the altered structure and conserved motifs of *EIN3/EIL* genes in broomcorn millet may therefore be associated with the observed changes in their expression levels.

### 4.3 The promoter regions of *EIN3/EIL* genes contain many cisregulatory elements associated with hormone response

The *cis*-Regulatory elements located in the promoter region of a gene are responsible for regulating gene expression (Nuruzzaman et al., 2014). In our investigation, we have identified a total of 10 *cis*-regulatory elements associated with hormone responses in the promoter regions of the broomcorn millet *EIN3/EIL* genes whereas there were only 5 in *Panicum hallii* (**Figure 4**). These elements play pivotal roles in mediating signal transduction of seven phytohormones (Li et al., 2019). Additionally, the abundance of ABA and SA response elements in broomcorn millet is significantly higher compared to *Panicum hallii*. Given the combined role of ABA and SA in plant growth, development, and response to environmental stimuli (Li et al., 2019), we speculate that this disparity may account for broomcorn millet’s enhanced environmental adaptability when compared to other species. With significant differences observed between broomcorn millet and *Panicum hallii* in terms of growth, development, and environmental adaptation, we propose that the type and quantity of *cis*-regulatory elements in the promoter region of the broomcorn millet *EIN3/EIL* gene may influence its ability to respond to environmental stresses, potentially reflecting the outcomes of natural selection to some extent.

### 4.4 The *EIN3/EIL* genes play important roles in plant growth and development and stress response

Tissue-specific transcriptome data can contribute to the prediction of the precise roles and functions of genes during plant growth and development. Among 15 *PmEIL* genes, the expression patterns of them in 8 tissues exhibited significant differences. *PmEIL15*, *PmEIL5*, *PmEIL6*, *PmEIL9*, *PmEIL2*, and *PmEIL10* exhibited high expression levels in mature seeds but relatively lower expression in other vegetative tissues suggesting the pivotal role in seed development. *PmEIL3* and *PmEIL8* displayed high expression levels in roots revealed the vital role in root growth since ethylene has been reported to exert a regulatory effect on the growth of root in plants. The tissue-specific variation of expression *EIN3/EIL* genes was also exist in other crops such as rice, maize, wheat and Brassica species (He et al., 2020; Jyoti et al., 2021). Meanwhile, transcriptome analysis showed that the *EIN3/EIL* genes exhibited unique expression profiles in response to different stresses. The expression of *PmEIL3* and *PmEIL8* was also significantly up-regulated under cold stress, which, combined with cis-acting element analysis, may be related to the presence of a large number of LTR elements in the promoter region, suggesting that *PmEIL3/8* may play an active role in plant growth and the defense of millet against cold stress. Interestingly, *PmEIL15* showed opposite expression trends in both salt and drought stresses in resistant/sensitive varieties, and we hypothesize that *PmEIL15* plays an important role in responding to abiotic stresses. Thus, the different expression patterns of *EIN3/EIL* genes in broomcorn millet showed important roles in growth and development and stress response.

## 5 Conclusion

Through our research, we have identified a total of 15 and 9 *EIN3/EIL* genes from broomcorn millet and *Panicum hallii* respectively. Analysis of these genes has revealed significant findings. Firstly, except for PmEIL1 and PmEIL15, all the *EIN3/EIL* genes of broomcorn millet had duplicate copies. Interestingly, the duplicates exhibited *Ka/Ks* < 1, indicating that they have undergone purifying selection throughout the course of evolution. Furthermore, most of the *EIN3/EIL* genes in broomcorn millet have lost their intron structure when compared to *Panicum hallii*. Additionally, the conservation of C-terminal motifs in broomcorn millet is lower compared to *Panicum hallii*. Within the promoter regions of *EIN3/EIL* genes in broomcorn millet, we have discovered a multitude of *cis*-regulatory elements associated with growth, development, hormone response, light response, and stress response. The number of hormone response elements, especially those related to ABA and SA, is higher in broomcorn millet than those in *Panicum hallii*. The RNA-sequencing based expression profiles of *PmEILs* in different growth and developmental stages and abiotic stresses suggesting their vital roles during the broomcorn millet life cycle. These findings provide valuable insights into the broomcorn millet *EIN3/EIL* gene family, enhancing our understanding of polyploidy and ethylene signaling pathways. They also serve as a valuable reference for future research endeavors in these areas.

## Data availability statement

The original contributions presented in the study are included in the article/**Supplementary Material**. Further inquiries can be directed to the corresponding author.

## Author contributions

MY: Writing – original draft, Software, Formal analysis. XS: Writing – original draft, Software, Data curation. JL: Writing – original draft, Formal analysis, Data curation. SW: Writing – original draft, Formal analysis, Data curation. MZ: Writing – original draft, Validation, Software. XD: Writing – original draft, Data curation. HW: Supervision, Writing – review & editing, Funding acquisition, Conceptualization.
