# Supplementary material for: Genome-wide identification and analysis of the EIN3/EIL gene family in broomcorn millet (Panicum miliaceum L.)
Source: Front Plant Sci. 2024 Aug 7;15:1440872. doi: 10.3389/fpls.2024.1440872 (PMC11335613; doi:10.3389/fpls.2024.1440872)
Supplement: Supplementary file 1 [file Table_1.docx]

Supplementary Table S1. Ka/Ks values of EIN3/EIL repeat gene pairs in broomcorn millet and Panicum hallii

| Duplicated gene pairs | Ka | Ks | Ka/Ks | Types of selection |
| --- | --- | --- | --- | --- |
| *PmEIL2-PmEIL9* | 0.034773568 | 0.05875196 | 0.591870767 | Purify selection |
| *PmEIL2-PmEIL10* | 0.286588555 | 0.437680874 | 0.654788848 | Purify selection |
| *PmEIL2-PmEIL12* | 0.327389949 | 0.504225239 | 0.649293062 | Purify selection |
| *PmEIL3-PmEIL6* | 0.321182218 | 1.391697804 | 0.230784454 | Purify selection |
| *PmEIL3-PmEIL5* | 0.305317206 | 1.309046199 | 0.233236387 | Purify selection |
| *PmEIL6-PmEIL5* | 0.031861379 | 0.083005414 | 0.383846999 | Purify selection |
| *PmEIL8-PmEIL3* | 0.014139691 | 0.059488299 | 0.237688601 | Purify selection |
| *PmEIL8-PmEIL6* | 0.321707116 | 1.294614189 | 0.248496517 | Purify selection |
| *PmEIL8-PmEIL5* | 0.304479139 | 1.220962775 | 0.249376267 | Purify selection |
| *PmEIL9-PmEIL10* | 0.286325488 | 0.433570152 | 0.660390219 | Purify selection |
| *PmEIL9-PmEIL12* | 0.343702213 | 0.569323813 | 0.603702506 | Purify selection |
| *PmEIL10-PmEIL12* | 0.049227653 | 0.060008926 | 0.820338842 | Purify selection |
| *PmEIL11-PmEIL14* | 0.012169967 | 0.059010559 | 0.206233713 | Purify selection |
| *PmEIL11-PmEIL4* | 0.25021433 | 0.559415842 | 0.447277877 | Purify selection |
| *PmEIL13-PmEIL7* | 0.322832866 | 0.403506967 | 0.800067637 | Purify selection |
| *PmEIL14-PmEIL4* | 0.226910299 | 0.538406317 | 0.421448063 | Purify selection |
| *PhEIL2-PhEIL8* | 0.320758225 | 0.51482088 | 0.623048206 | Purify selection |
| *PhEIL4-PhEIL7* | 0.329056847 | 1.156935257 | 0.284421142 | Purify selection |
| *PhEIL6-PhEIL1* | 0.22837012 | 0.548747139 | 0.416166398 | Purify selection |
